# Cochlear implants in a low-income country: Brazilian public health system (SUS) - a longitudinal analysis since the beginning

**DOI:** 10.1016/j.bjorl.2020.12.001

**Published:** 2020-12-26

**Authors:** Carla Valença Daher, Fayez Bahmad

**Affiliations:** Universidade de Brasília (UnB), Faculdade de Ciências de Saúde, Programa de Pós-Graduação em Ciências da Saúde, Brasília, DF, Brazil

Cochlear implants (CI) have been indicated as a treatment option for adult and pediatric patients with severe to profound hearing loss and have been offered by the Brazilian public healthcare system, the Unified Health System (SUS), since 1993 when the first regulation was proposed by the government.

From 1999 to 2019, several regulations were issued, all of which were related to CI in the SUS and aimed to standardize the provided care and improve recording clinical information regarding CI in the SUS information systems.

Of the regulations published during this period Ministry of Health Ordinance no. 2.776/2014 stands out. It focuses on providing comprehensive care for hearing-impaired individuals with indications for CIs (unilateral and bilateral) and bone-anchored hearing aids (unilateral and bilateral) and extends from clinical diagnosis through surgery to periodic follow-ups and auditory habilitation or rehabilitation, as established in the general guidelines for the epecialized care of individuals with hearing impairment in the SUS.^1^ It establishes that this care should be structured as an outpatient modality, with clinical and audiological evaluations, follow-ups and speech therapy rehabilitation, with an in-hospital modality, regarding the performance of surgeries and pre- and postoperative follow-ups.

Surgical treatment is just one action within the entire line of care of people with hearing impairment. It is indicated only in patients with bilateral sensorineural hearing loss of severe to profound degree, who meet the indications provided for in the guidelines.

Great advances have been achieved with this new standardization, such as: updating the clinical indications of unilateral cochlear implant, inclusion of bilateral cochlear implant indications and bone-anchored hearing aid (unilateral and bilateral), and costing for the maintenance of the external component of cochlear implant by the Ministry of Health and extending the warranty period.

Another major advance was consolidated with the publication of Ordinance No. 2,161, of 07/17/2018, which included the exchange of the speech processor in the SUS reimbursement system, according to technical criteria.

According to data from Datasus, National Health Establishments Registry System (*Sistema de Cadastro Nacional de Estabelecimento de Saúde, SCNES*), there are currently 33 services accredited to perform CI surgery in all regions of Brazil. The highest concentration of these services is found in the Southeast region (45%), which is the region with the highest number of hospitals, medical and training centers offering rehabilitation with CI since 1990.

It is also observed that, although there are accredited services in all regions of the country, many states still provide this care through out-of-state treatment (*tratamento fora de domicílio - TFD*).^2^ TFD involves an agreement among states in which a state sends its residents to the nearest centers to undergo CI rehabilitation.

It is important to note that the existence of care void is a reality observed not only in this area but in most specialties that require high-complexity services, characterized by sophistocated technology, high costs and the need for specific physical infrastructure and specialized health care, which are not available in all regions of our country.^3^

Regarding the characteristics of the health facilities, it was observed that 45% of the services are municipally managed, 85% are general hospitals, and 45% are nonprofit (philanthropic) charities. This predominance of private philanthropic facilities under municipal management for the provision of medium- and high-complexity SUS services is historical and involves regional governance arrangements that include the prioritization of regional strategies for intergovernmental negotiation, investment, planning and the expansion of the service network to address health inequalities.^4^

It was also found that 85% of the accredited facilities are general hospitals, which allow greater integration of medical professionals (otorhinolaryngologists, general practitioners, neuropediatric team, neurologists, pediatricians, radiologists, cardiologists, anesthesiologists, plastic surgeons and geneticists), nonmedical professionals (speech therapists, nurses, psychologists and social workers) and support services, such as nutrition, pharmacy, radiology and hemotherapy. This concentration of facilities in general hospitals is considered a positive factor because this patient population requires the integration of multiple services.

Specialized health care generally involves high levels of technology and care (outpatient and in-hospital) with high costs, and this is not different for CI. A total of 38 CI-related procedures beyond the procedure of CI by itself can be identified in the SUS procedures reimbursement system, and 10.5% of them are classified as high complexity. Regarding surgical procedures, 44.7% are classified as medium complexity, and 44.7% do not require the classification of complexity, which is the case for orthoses, prostheses and special materials (OPM). Regarding the level of care, 78.9% of the procedures are performed on an outpatient basis, 15.8% are performed in-hospital, and 5.3% can be performed on either an outpatient or inpatient basis. These data show that just over 10% of procedures are high complexity and high cost (i.e., surgeries) and are performed by otorhinolaryngologists in hospital. Postsurgical care (the activation of electrodes, mapping and telemetry, hearing evaluation and speech therapy) is considered moderately complex, is performed on an outpatient basis by speech therapists, and is essential for the proper use and better performance of CI.

The funding of health actions, which is currently the responsibility of federal, state and municipal government is available for medium- and high-complexity health actions and services in outpatient and in hospital settings. It is organized in two components that involve fund transfers from the federal government. These components comprise the Medium and High Complexity Outpatient and Hospital Funding Limit Component (*Limite Financeiro da Média e Alta Complexidade Ambulatorial e Hospitalar – MAC*) and the Strategic Actions and Compensation Fund (*Fundo de Ações Estratégicas e Compensação – FAEC*).

In hearing rehabilitation with CI, most procedures (65.8%) are funded by the FAEC, and 34.2% are funded by the MAC ceiling. This is due to the incorporation of new procedures by the SUS reimbursement system in 2014.

According to the Datasus database,^5^ between 2000–2019, 10.427 CI surgeries were performed in Brazil, and 86,076 post-surgical follow-ups with a gradual increase over the years compatible with the increment of number of accredited services.

Regarding investments in CI surgery during the same period, there was a gradual increase compatible with the number of services that have been accredited over the years, supported by an accumulated investment of R$ 476,728,866.88 (Brazilian reais).The mean cost of CI surgery was R$ 45,720.62 (Brazilian reais).

In the analysis of invested resources, the years 2003 and 2006 are noteworthy due to the large difference in the percentage of spending from one year to the next. This occurred because of changes in the cost of the CI prosthesis, which is based on variations in the US dollar, which had an increase of 85% in 2002. There was also a decrease in spending from 2005 to 2006 as a result of the 79% reduction in the cost of CI prostheses after an economic study conducted by the Ministry of Health.

It is noteworthy that the postsurgical follow-up of CI users is not limited to monitoring the electronic device after surgery but rather is a continuous and complete process of hearing habilitation, especially for children.

Thus, throughout the 30 years of the SUS, the Ministry of Health has issued regulations, developed policies, regulated actions and services and provided resources in the area of hearing health, including the rehabilitation of hearing loss in patients using Cis, however, it is still necessary to strengthen actions of promotion, prevention, identification and early intervention in hearing loss, in addition to improving strategies for the implementation of post-surgical follow-ups of users, in accordance with the standards, principles and functioning of the SUS.

## Conflicts of interest

The authors declare no conflicts of interest.
